# CREPT Promotes Melanoma Progression Through Accelerated Proliferation and Enhanced Migration by RhoA-Mediated Actin Filaments and Focal Adhesion Formation

**DOI:** 10.3390/cancers12010033

**Published:** 2019-12-20

**Authors:** Hui Liu, Ann L. B. Seynhaeve, Rutger W. W. Brouwer, Wilfred F. J. van IJcken, Liu Yang, Yinyin Wang, Zhijie Chang, Timo L. M. ten Hagen

**Affiliations:** 1 Laboratory of Experimental Oncology, Department of Pathology, Erasmus University Medical Center, 3015 GD Rotterdam, The Netherlands; 2 Center for Biomics, Erasmus University Medical Center, 3015 GD Rotterdam, The Netherlands; 3 State Key Laboratory of Membrane Biology, School of Medicine, National Engineering Laboratory for Anti-Tumor Therapeutics, Tsinghua University, Beijing 100084, China

**Keywords:** melanoma, CREPT, proliferation and migration, cytoskeleton organization, RhoA activation

## Abstract

Melanoma is one of the most aggressive cancers, and patients with distant metastases have dire outcomes. We observed previously that melanoma progression is driven by a high migratory potential of melanoma cells, which survive and proliferate under harsh environmental conditions. In this study, we report that CREPT (cell-cycle related and expression-elevated protein in tumor), an oncoprotein highly expressed in other cancers, is overexpressed in melanoma cells but not melanocytes. Overexpression of CREPT stimulates cell proliferation, migration, and invasion in several melanoma cell lines. Further, we show that CREPT enhances melanoma progression through upregulating and activating Ras homolog family member A (RhoA)-induced actin organization and focal adhesion assembly. Our study reveals a novel role of CREPT in promoting melanoma progression. Targeting CREPT may be a promising strategy for melanoma treatment.

## 1. Introduction

Malignant melanoma, which derives from melanocytes and mostly arises in the skin, is characterized as a highly metastatic tumor. In the past 30 years, the incidence of cutaneous melanoma has grown rapidly [1]. Around 300,000 new cases of melanoma were reported worldwide in 2018, and the incidence of melanoma was ranked fifth of all cancers in the United States [1,2]. Due to the rapid progression and metastasis, ~5% of patients present distant metastasis at the initial diagnosis [3]. Although immunotherapy or targeted therapy drugs yield notable clinical benefits, increasing five-year overall survival to 35% to 50%, a great proportion of metastatic melanoma patients remain unresponsive to treatments [4,5,6]. For this reason, finding the cause of melanoma progression remains crucial to further improve therapeutic strategies.

Metastases, in which tumor cells disseminate to a distant site, are the main cause of cancer deaths [7]. Several steps can be identified during the metastatic process, which demands certain cell characteristics. Tumor cells dissociating from primary sites must have the ability to migrate and invade through the basement membrane and extracellular matrix (ECM). To pass through tissues, tumor cells change their morphology and stiffness to fit in and interact with surrounding ECM structures [8]. These morphological changes, like cell polarization and formation of membrane extensions, are inseparable with filamentous actin and related cell-matrix attachments [9,10,11]. Integrins, which are transmembrane receptors and couple the cell to ECM components, regulate focal contact assembly and cell migration in melanoma [12]. Rho GTPase signaling contributes to cell movement by influencing cytoskeleton reorganization [13,14]. After the process of intravasation and extravasation, metastatic tumor cells travel to a secondary site, adapt to the local microenvironment for survival, proliferate, and develop a vasculature to establish a new tumor [15,16]. Melanoma cells are also able to sustain the survival of endothelial cells under restrictive conditions [17]. The strong angiogenic potential contributes to fast metastasis of melanoma.

Regulation of nuclear pre-mRNA domain-containing (RPRD) proteins are identified as novel RNA polymerase II (RNAPII)-interacting proteins, which are evolutionarily conserved and ubiquitously expressed in human tissues. All three RPRDs (including RPRD1A, RPRD1B, and RPRD2) contain C-terminal domain (CTD) interaction domains. RPRD1A and RPRD1B associate with RNAPII phosphatase directly and interact with CTD heptapeptide repeats to recruit RNAPII for dephosphorylation of phosphor-S5 [18,19] Recently, an enhanced expression of CREPT, also known as RPRD1B or C20orf77, is reported in many human tumors, including gastric cancer, endometrial cancer, and colorectal cancer [20]. CREPT promotes cell proliferation and tumorigenesis by regulating the cell cycle process by enhancing transcription of Cyclin D1, Cyclin B1, Cyclin-dependent kinase 4 and 6 (CDK4 and CDK6) [20,21,22]. CREPT also shows an oncogenic potential as a transcription co-activator of the β-catenin·TCF4 (transcription factor 4) complex to promote the transcriptional activity of the Wnt signaling pathway [23]. Besides, it is reported that a new CREPT/c-myc/CDC25A pathway may result in cell growth and migration promotion [24]. While the function of CREPT has been characterized to some degree in several cancers, the role in cell migration and invasion is less known, and the contribution of CREPT in melanoma tumorigenesis has not been studied.

Here, we show the differential expression of CREPT in melanoma cells and melanocytes, and evaluate the oncogenic potential by assessing the association with cell proliferation, migration, and invasion. Moreover, we find that CREPT influences cell migration by regulating RhoA-related actin filaments’ polymerization and focal adhesion formation. Our results demonstrate that CREPT functions as an oncoprotein in melanoma and modulates cell migration and invasion.

## 2. Results

### 2.1. Differential Expression of CREPT in Melanoma Cells and Melanocytes

**Figure 1:**
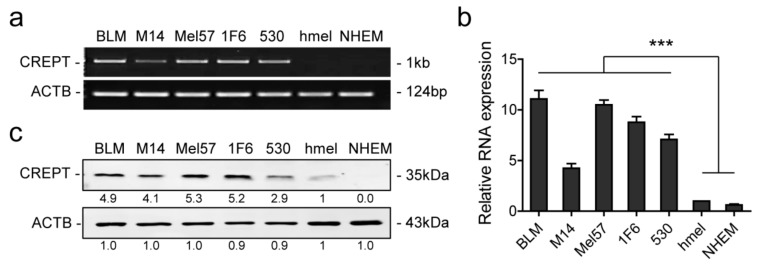
CREPT is highly expressed in melanoma cells compared to melanocytes. (**a**) Representative pictures of RT-PCR to show CREPT expression in all cell lines. (**b**) Quantitative PCR is displayed as mean ± SEM and statistical analysis was conducted with hmel as the control group. (**c**) Representative pictures of western blotting showing endogenous CREPT protein expression in all cell lines. Beta-actin (ACTB) is used as a loading control. Numbers under the bands show the mean of the intensity ratios standardized to hmel. *** *p* < 0.001. All experiments were repeated at least three times.

To investigate whether CREPT plays a role in melanoma, the expression in different cell lines was examined at both the mRNA and protein level. PCR, qPCR, and western blotting results show high expression of CREPT in all five melanoma cell lines (BLM, M14, Mel57, 1F6, 530) and low or absent expression in two melanocyte lines (hmel and NHEM) (Figure 1). These results suggest that CREPT is elevated in melanoma but is low in normal melanocytes.

### 2.2. Depleted or Enforced Expression of CREPT Influences Melanoma Cells’ Proliferative Capacity

**Figure 2:**
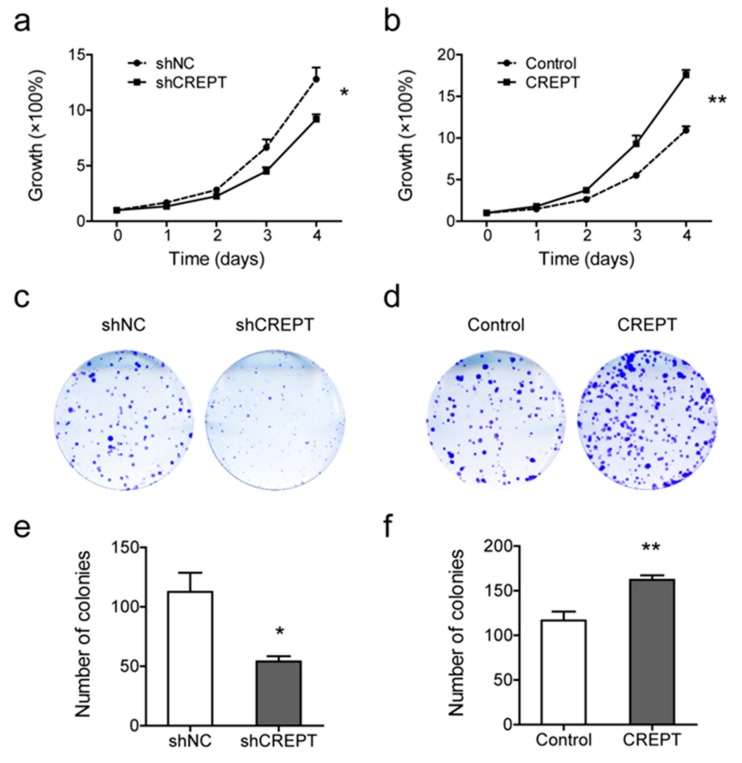
Depletion or overexpression of CREPT affects BLM cell proliferation. Knockdown of CREPT (shCREPT) is compared with control (shNC)”, and overexpression of CREPT (CREPT) is compared with control (Control). (**a**,**b**) In vitro cell growth rates of BLM cells with different CREPT expressions are shown as mean ± SEM of three independent experiments. (**c**,**d**) Representative pictures of the colony formation status in cell lines with distinct CREPT expression. (**e**,**f**) Colony numbers were measured in Fiji and data are shown as mean ± SEM of four independent experiments. * *p* < 0.05, ** *p* < 0.01.

**Figure 3:**
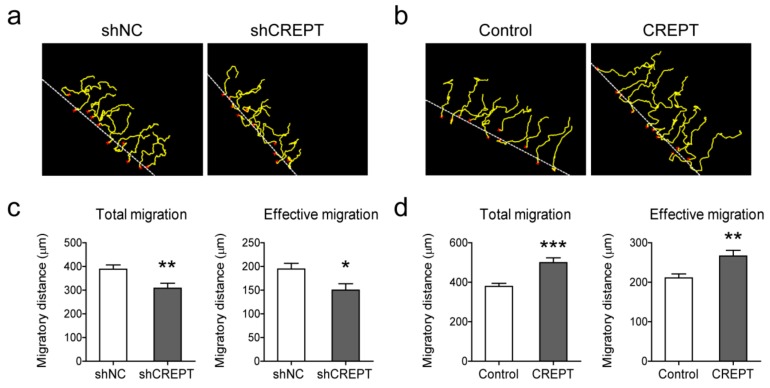
Effect of CREPT expression on BLM cell migration. Knockdown of CREPT (shCREPT) is compared with control (shNC)”, and overexpression of CREPT (CREPT) is compared with control (Control). (**a**,**b**) Representative pictures of cell trajectories in 24 h. Dotted white lines indicate the migration front at *t* = 0, red crosses indicate cells selected for tracking, and yellow lines represent single cell trajectories representing the total migration. (**c**,**d**) Migratory parameters were quantified and calculated of 30 cells in each individual experiment. Total migration (μm) is the total distance every cell moves in 24 h. Effective migration (μm) is the net displacement from 0 to 24 h. Data represent mean ± SEM of three independent experiments. * *p* < 0.05, ** *p* < 0.01, *** *p* < 0.001.

**Figure 4:**
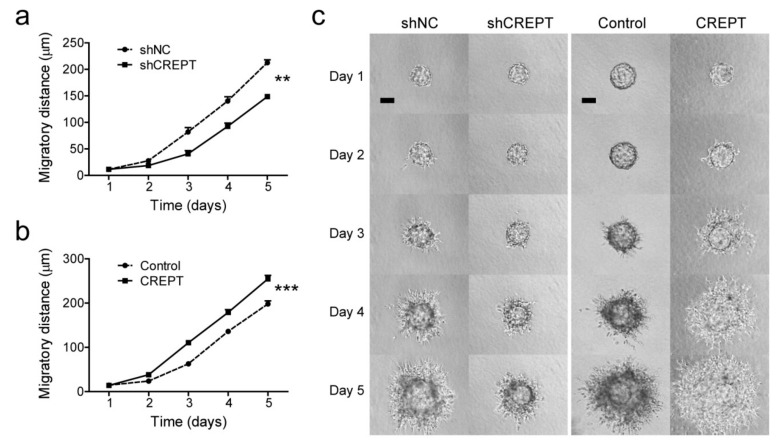
The invasive capacity of BLM cells is affected by modified CREPT expression. Microcarrier beads covered with cells were embedded in collagen gel and cell dispersion into the matrix was photographed every day for evaluation. In total, 10 to 15 beads per group were included in each independent experiment, and dispersion distances were quantified and analyzed as shown in panels (**a**,**b**). Data is depicted as mean ± SEM of three independent experiments. ** *p* < 0.01, *** *p* < 0.001. (**c**) Representative pictures of cell dispersion over time for different cell lines. Scale bars, 100 µm.

**Figure 5:**
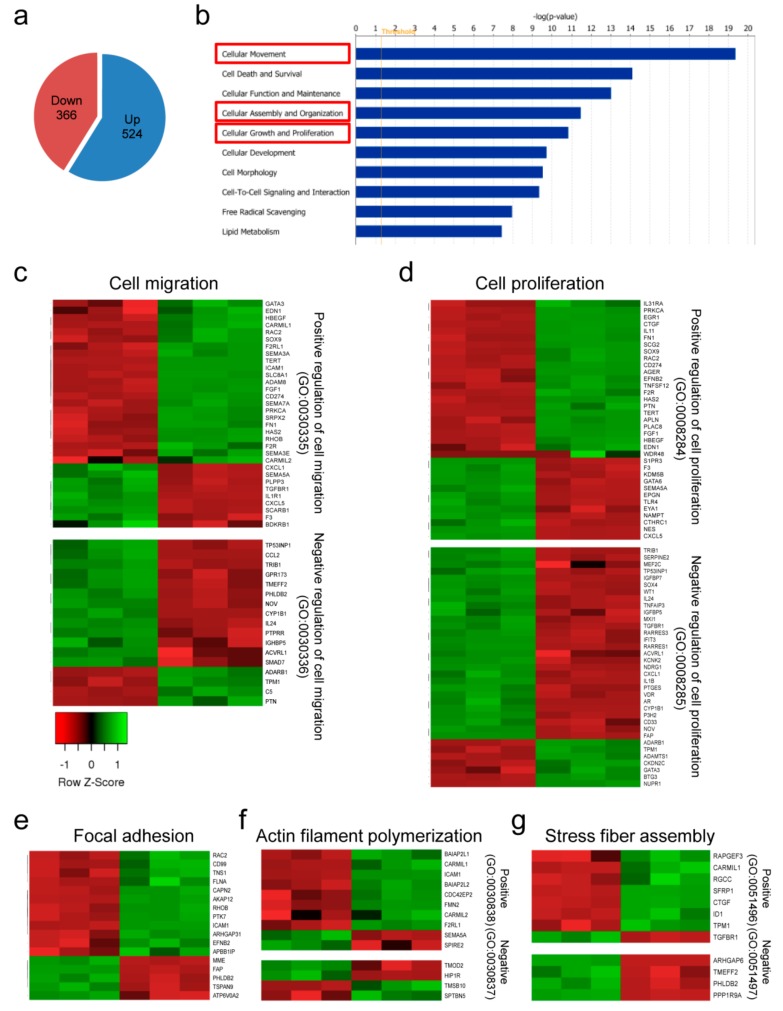
Global gene changes in melanoma cells with differential CREPT expression in BLM cells. (**a**) The pie chart shows the global up- and downregulated transcript numbers. (**b**) The top 10 most correlated molecular and cellular functions. The x-axis displays the -log of significance. (**c**–**g**) Heat maps of gene ontology (GO) classification in which genes are significantly changed according to the depletion of CREPT. Samples are displayed in the order of BLM-shCREPT (*n* =3, left) and BLM-shNC (*n* = 3, right). Heat maps were made based on z-scores, which indicate the data dispersion from the mean. (**c**) Altered genes in the GO term “regulation of cell migration”. The majority of the downregulated genes involved in positive regulation of cell migration and most upregulated genes in the negative effect were observed in CREPT-depleted samples. Similar observations were made in the “regulation of cell proliferation” (**d**), GO:0005925“focal adhesion” (**e**), “actin filament polymerization” (**f**), and “stress fiber assembly” (**g**).

**Figure 6:**
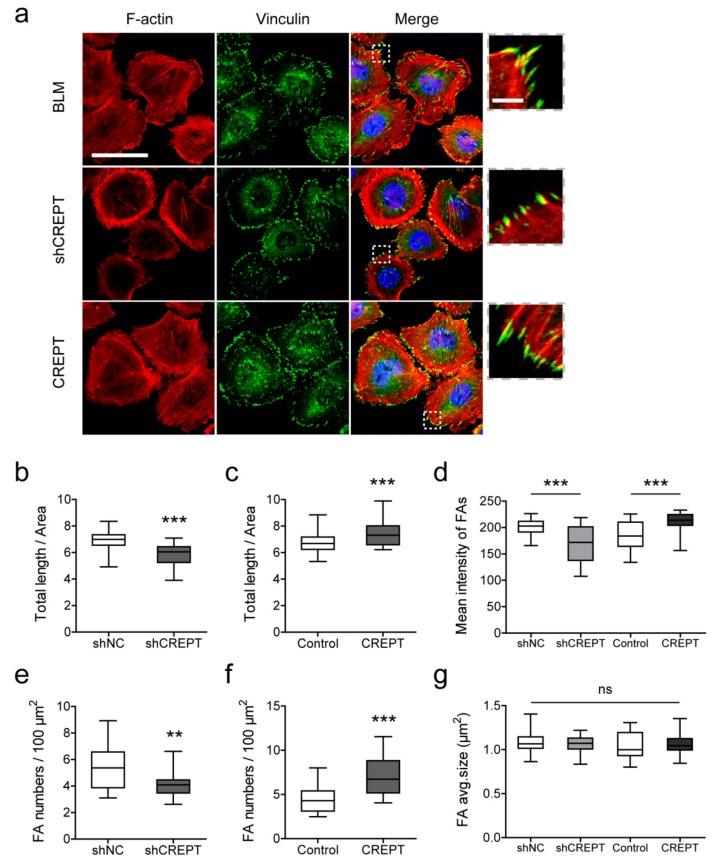
Immunofluorescence staining to visualize F-actin and focal adhesions in BLM cells. (**a**) Representative pictures of non-transfected BLM, BLM with low CREPT levels (BLM-shCREPT), and BLM with high CREPT levels (BLM-CREPT). Images in dotted squares are magnified to see focal adhesions at the end of actin filaments. Scale bar in large images, 50 µm; scale bar in zoomed in panels, 5 µm. After image processing and quantification, the total length of actin filaments and focal adhesion numbers in each cell were measured. To eliminate bias against different cell sizes, data were standardized by the area unit and are shown in boxplots. (**b**,**c**) The total length (µm) of actin filaments per area (µm^2^). (**d**) Mean florescence intensity of focal adhesions in individual cells. (**e**,**f**) Focal adhesion numbers per 100-µm^2^ area. (**g**) Average size of focal adhesions in individual cells. Data in panels (**b**–**g**) represent 40 cells/measurements in three independent experiments. ** *p* < 0.01, *** *p* < 0.001; ns, not significant.

**Figure 7:**
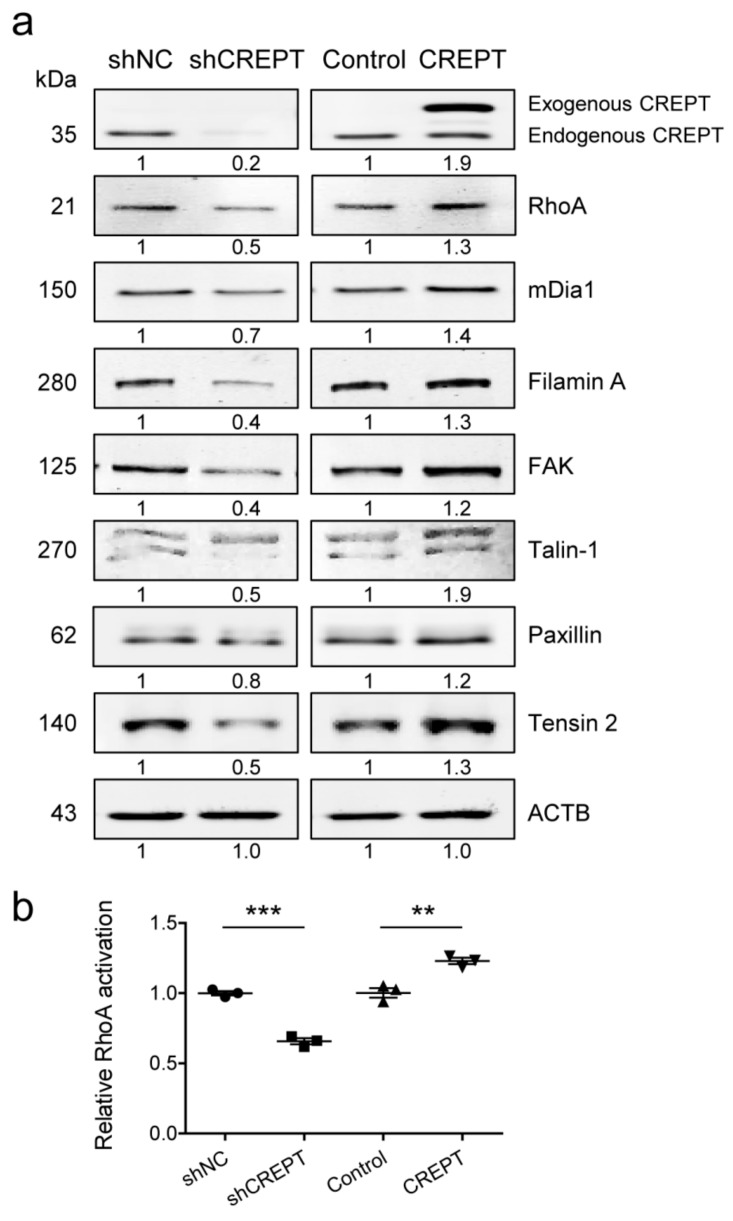
CREPT expression influences actin filament/focal adhesion formation-related signaling molecules in BLM cells. (**a**) Western blotting was performed to evaluate expression of RhoA, Diaphanous related Formin 1 (mDia1), FilaminA, focal adhesion kinase (FAK), Talin-1, Paxillin, Tensin 2. Beta-actin (ACTB) was used as the loading control. Representative results are shown followed by mean protein expression values from four independent experiments. (**b**) Activated RhoA levels were quantified with small GTPase activation assay, and relative values are shown as mean ± SEM of three independent experiments. ** *p* < 0.01, *** *p* < 0.001.

To reveal the function of CREPT in melanoma, BLM cells were stably transfected with CREPT coding sequence plasmid and empty vector (shown as “CREPT” and “Control”, respectively, in Figure 2, Figure 3, Figure 4, Figure 5, Figure 6 and Figure 7) for enhanced expression, and with plasmids inserted with short hairpin RNA (shRNA) against CREPT and scrambled shRNA (shown as “shCREPT” and “shNC”, respectively, in Figure 2, Figure 3, Figure 4, Figure 5, Figure 6 and Figure 7) for depleted expression (Figure S1). We performed SRB assays to evaluate cell proliferation rates. The results show that depletion of CREPT led to a decreased growth rate, and reciprocally, overexpression of CREPT promoted cell proliferation significantly (Figure 2a,b). To verify CREPT functions in other melanoma cells, we also selected M14 cells for CREPT overexpression and MEL57 cells for CREPT depletion because M14 had a relatively low endogenous CREPT level and MEL57 had a high level (Figure 1 and Figure S1). SRB assays show similar trends of CREPT-regulated cell growth rates to BLM cells (Figure S2a). To address the role of CREPT in the malignant feature of melanoma, we examined the colony formation ability of CREPT-modified BLM cells. The results show that colonies of CREPT-depleted cells were reduced to 54.0 ± 4.4 per well as compared to 112.5 ± 16.2 per well for non-depleted cells (*p* < 0.05) while CREPT-overexpressed cells formed more colonies than control cells (162.3 ± 4.9 vs. 116.8 ± 9.9 per well, *p* < 0.01) (Figure 2c–f). These results suggest that proliferative and clonogenic potentials of melanoma cells are, at least in part, dependent on CREPT expression.

### 2.3. CREPT Promotes Melanoma Cell Migration and Invasion In Vitro

To address the effect of CREPT on cell migration, migratory profiles of melanoma cells were evaluated by a ring-barrier migration assay. Cell movement was monitored in reduced-serum media (DMEM supplemented with 1% FBS) to diminish the impact of cell proliferation. We followed cell trajectories for 24 h and measured total and directional distances. Total migration indicates the capacity of cell motility, and effective migration shows net displacement, implying persistent directionality of cell migration. BLM cells with CREPT depletion (BLM-shCREPT) show significantly reduced total migration distances and net displacement (Figure 3a,c) while overexpression of CREPT increased the total migration and effective migration (*p* < 0.01) (Figure 3b,d). Similar results were observed in MEL57 and M14 cells (Figure S2b). These results demonstrate that CREPT facilitates melanoma cell motility and migration.

The migration potential in two dimensions was tested with cells attached to a surface, where cells can only have contact with the extracellular matrix (ECM) on the surface side. To investigate the effect of CREPT on three-dimensional migration or invasion capacity, we used microcarrier beads as supporting cores to grow spheroids and monitor cell invasion by embedding spheroids into a 3D matrix. This assay enables cell–ECM interaction on all sides of the cell during movement, resembling a more realistic microenvironment. In 5 days, BLM-shCREPT cells displayed a decreased invasive distance (148.7 ± 4.1 µm) in comparison to BLM-shNC cells (212.7 ± 5.3 µm, *p* < 0.01) (Figure 4a,c). On the contrary, overexpression of CREPT led to an faster invasion into the matrix than control BLM cells (255.0 ± 7.7 µm vs. 198.1 ± 7.1 µm, *p* < 0.001) (Figure 4b,c). The results were confirmed with MEL57 and M14 cells (Figure S2c). These data illustrate that CREPT stimulates the invasive process of melanoma cells in the 3D collagen matrix.

### 2.4. Global Gene Expression Profiling Based on CREPT Expression Modification

To understand the molecular mechanisms of CREPT-induced changes in cell behavior, we performed whole transcriptome sequencing using BLM-shCREPT cells and BLM-shNC cells. In total, 366 downregulated and 524 upregulated transcripts were obtained when comparing BLM-shCREPT to BLM-shNC samples (Figure 5a). Gene molecular and cellular function analysis indicated that these genes were enriched in functions “cellular movement”, “cellular assembly and organization”, and “cellular growth and proliferation” (Figure 5b). Further, to visualize gene changes in categories, we clustered significantly altered genes and showed the results in heat maps. In correlation with our experimental observations on CREPT-regulated cell proliferative and migratory capacity, we first checked GO:0030334 “regulation of cell migration” and GO:0042127 “regulation of cell population proliferation”, which represent the process modulating the frequency, rate, or extent of cell migration and cell proliferation, respectively. Genes involved in positive regulation of cell migration and proliferation were mostly downregulated (23/32, 72% in migration; 23/35, 66% in proliferation) in BLM-shCREPT samples while genes involved in negative regulation of cell migration and proliferation were mainly upregulated (13/17, 76% in migration; 28/35, 80% in proliferation) (Figure 5c,d). The results support the biology profiling that CREPT improved melanoma cell proliferation and migration. Apart from the functions we observed in the experiments, the gene expression profiling also showed a link between CREPT expression and actin cytoskeleton organization, which is essential for cell migration. To maintain the actin skeleton structure, globular actins firstly polymerize to filaments. Actin filaments together with myosin II and other cytoskeletal proteins assemble to form stress fibers working as contractile structures. These actin bundles terminate at the cell surface, where focal adhesion assemblies anchor the cell to the extracellular matrix [25]. Therefore, we zoomed in on the expression profile of genes involved in focal adhesion, actin polymerization, and stress fiber assembly. The RNA-Seq data show that the major cluster of genes in BLM-shCREPT samples are downregulated in “focal adhesion” (12/17, 71%), “positive regulation of actin polymerization” (8/10, 80%), and “positive regulation of stress fiber assembly” (7/8, 88%) (Figure 5e–g). Taken together, the gene profiling confirms the experimental results that CREPT promotes cell proliferation and migration, and gives directions for further examination of molecular mechanisms.

### 2.5. CREPT Regulates Actin Filament Polymerization and Focal Adhesion Formation

According to global gene expression profiling, we identified the effect of CREPT on the actin cytoskeleton and focal adhesion-related biological processes. To visualize actin filaments and focal adhesions at the cellular level, immunofluorescence staining was performed against F-actin and vinculin (Figure 6a). The quantified data show significantly shorter lengths of filaments and fewer focal adhesion numbers in BLM-shCREPT cells than those in BLM-shNC cells (Figure 6b,e). On the contrary, BLM-CREPT cells demonstrated more filaments and focal adhesions than control cells (*p* < 0.001) (Figure 6c,f). In addition to focal adhesion numbers, we also quantified the mean size of focal adhesions but found no significant changes in size based on CREPT differential expression (Figure 6g). Measurements of the mean florescence intensity of focal adhesions indicated the vinculin expression level as a component of the focal adhesion complex. We detected a decrease of vinculin in low CREPT-expressing cells and an increase of vinculin in high CREPT-expressing cells (Figure 6d). These results illustrate that CREPT promotes actin filament polymerization and focal adhesion formation in melanoma cells.

### 2.6. CREPT Influences Focal Adhesion Signaling-Related Molecules

To interpret the difference in actin filament polymerization and focal adhesion formation, gene expression was examined on the protein level by western blotting. CREPT protein was detected to show an efficacy of depletion and overexpression. For BLM-CREPT cells, exogenous CREPT protein was labelled with an HA (hemagglutinin)-tag, which made the protein size approximately 36 to 37 kDa, showing a band above endogenous CREPT in western blotting. We observed that expression of actin polymerization-related genes (RhoA, mDia1) and focal adhesion-related genes (FilaminA, FAK, Talin-1, Paxillin, Tensin 2) correlates with the expression of CREPT (Figure 7a, Figure S3.1 and Figure S3.2). We verified with the small GTPase activation assay that activation of RhoA is regulated by CREPT (Figure 7b). These results demonstrate that CREPT activates the RhoA/mDia1 axis and increases the expression of focal adhesion-related proteins.

### 2.7. The Role of RhoA on Actin Filaments and Cell Migration

**Figure 8:**
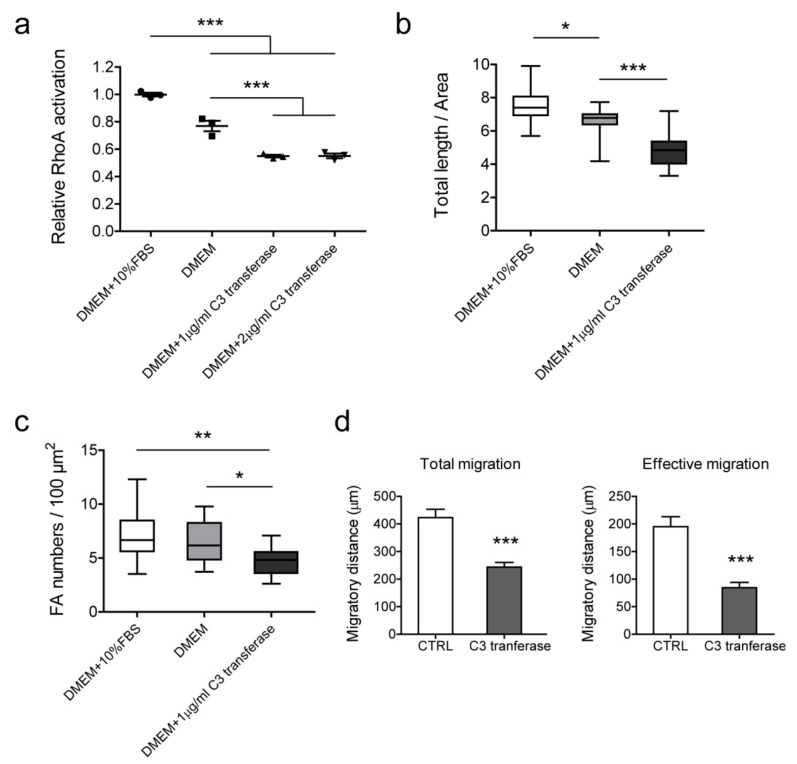
The effect of Rho inhibition on actin filaments, focal adhesion, and cell migration in BLM cells. (**a**) Cell-permeable C3 transferase was used in serum-free medium to inhibit Rho activation. BLM cells were treated with different conditions for 2 h and lysates were collected for RhoA activation assay. Standardized activation levels are shown as mean ± SEM of three independent experiments. Immunofluorescence staining was performed to evaluate changes in actin filaments (**b**) and focal adhesions (**c**). Data of 16 cells were measured and statistically analyzed from three independent experiments. (**b**) Total length (µm) of actin filaments of per cell area (µm^2^). (**c**) Focal adhesion numbers of per cell area (100 µm^2^). (**d**) Cell migration was determined by ring-barrier migration assay. BLM cells were treated with serum-free DMEM as a control and 1 μg/mL C3 transferase in DMEM for Rho inhibition. After the 2-h treatment, medium were refreshed with DMEM + 1% FBS and time-lapse imaging was performed for 24 h. Data represent mean ± SEM of three independent experiments. * *p* < 0.05, ** *p* < 0.01, *** *p* < 0.001.

To decipher whether the influence of CREPT on cell migration and formation of actin filaments/focal adhesions correlated with RhoA activation, we inactivated Rho proteins in BLM cells using C3 transferase. The results show that C3 transferase inhibited Rho activity to 50% at a dosage of 1 to 2 μg/mL (Figure 8a). As the high dosage did not increase the efficiency, we treated cells with 1 μg/mL C3 transferase to observe actin filament formation and evaluate cell migration ability. The immunofluorescence staining results show a significant decrease of the total length and focal adhesion numbers of actin filaments in Rho-inhibited cells (Figure 8b,c). Intriguingly, cell migratory capacity in 2D was significantly inhibited by Rho inactivation (Figure 8d). These results link the CREPT-regulated phenotype with RhoA activation, and demonstrate that CREPT influences cell migration, and actin filaments/focal adhesion formation, through RhoA activation.

## 3. Discussion

Cell migration and metastases formation are crucial for malignant progression, which leads to increased mortality in cancer patients. Recent studies revealed the oncogenic potential of CREPT expression in multiple cancer types, including correlation with poor prognosis, induction of cell proliferation in vitro, and tumorigenesis in vivo [26,27,28]. Knockdown of RPRD1B increased cell sensitivity to treatment in endometrial adenocarcinoma, which suggests a therapeutic potential for targeting CREPT [21]. However, the role of CREPT in regulating cell migration and invasion is rarely discussed. As melanoma is known for high aggressiveness and strong potential of metastasis, in this study, we examined the contribution of CREPT on the migration and motility in melanoma cells. PCR and western blotting data show high transcription and protein level of CREPT in melanoma cells, and low or absent expression in human melanocytes. Melanocytes were immortalized for in vitro culture and served as the non-tumor control here. The findings demonstrate a differential expression of CREPT in melanoma and non-tumor cells, indicating that CREPT may function as an oncogene and may contribute to melanoma progression.

Enhanced cell growth is a typical feature of cancer. A previous study showed that CREPT promotes cell growth by regulating cell cycle-related cyclins and kinases [20]. It was also reported that CREPT regulates colorectal cancer cell proliferation and tumorigenesis through Wnt/β-catenin signaling [29]. In addition to improving proliferation, CREPT also reduces cell apoptosis by regulating the ROS/p53 pathway, and thus increases tumor growth [30]. Consistent with the published data, our results indicate that a high level of CREPT facilitates cell division and colony formation in melanoma. Another feature of cancer is metastasis. Increasing cell motility provides more possibilities for tumor metastasis. A study in gastric cancers revealed that CREPT increases cell migration by regulating E-cadherin, vimentin, N-cadherin, and matrix metalloproteinase 1 (MMP-1) expressions [30]. To assess the effect of CREPT on cell motility, we examined cell migration in 2D conditions and invasive capacity in 3D matrices, which mimiced an in vivo environment. Since maintaining living cells inevitably causes cell division during migration assays, we tracked single cell movement to reveal cell motility. 2D and 3D migration results indicate that CREPT induces melanoma cell motility, migration, and invasion. The low expression level of CREPT in melanocytes provides an interesting possibility to study whether forced expression of CREPT in these cells would inflict a malignant transformation, which is currently under investigation in our group. Taken together, our findings show evidence at the cellular level that CREPT promotes tumorigenesis-related biological processes, suggesting a non-negligible role of CREPT in the progression of melanoma.

Migrating cells have a polarized morphology with protrusions like lamellipodia and filopodia, which are the leading part in the direction of movement. These protrusions are driven by actin polymerization and filament assembly [31]. Focal adhesions connect actin cytoskeleton and extracellular matrix at the cell membrane, allowing actin networks to pull the cell forward [32]. The transcriptome sequencing analysis indicates that genes involved in the processes of “focal adhesion”, “actin polymerization”, and “stress fiber assembly” are regulated by CREPT. Further, immunofluorescence staining shows a CREPT-related enhancement of actin filaments and focal adhesion formation, which gives a subcellular interpretation of CREPT-regulated migration. Assembly and disassembly of focal adhesions dynamically occur during cell migration but are regulated by different mechanisms. Integrins aggregate and recruit adaptor proteins, like talin, tensin, and paxillin, to form adhesions connecting ECM structures and actin filaments. On the other hand, the disassembly process is triggered by microtubule extensions to focal adhesions [33]. In our study, the result of the dynamic equilibrium of focal adhesion formation. Focal adhesion protein levels and size were reported to be closely related to cell migration [34] while in this study, we did not observe a significant change in the mean size of focal adhesions with respect to CREPT differential expression. This result may be explained by the biphasic relationship between focal adhesion size and cell migration speed. It is reported that the mean size of focal adhesions is highly predictive of cell migration speed, however, the correlation is non-linear. Increased migration speed is accompanied by an increase in focal adhesion size until it plateaus, after which growing focal adhesions are associated with reduced migration speed. [35]. This phenomenon is also related to adhesion maturation and turnover. How and to what extent CREPT influences the focal adhesion dynamic process is still under investigation in our group.

Small GTPase protein RhoA is involved in the focal adhesion signaling pathway (KEGG:hsa04510) and regulates actin cytoskeleton organization. RhoA can activate Rho effector mDia1, which binds directly to the fast-growing barbed end of actin filaments and facilitates actin nucleation and elongation [36,37]. In this study, we observed that CREPT upregulates RhoA activation and mDia1 expression. The results indicate a CREPT-RhoA-mDia1 signaling axis, which leads to actin organization regulation. One limitation is that we show an indirect interaction of CREPT and RhoA. How CREPT activates RhoA remains unknown, which would be an interesting question to study. To verify the role of RhoA in CREPT-induced cellular processes, we inhibited RhoA activation and consequently, actin filament/focal adhesion formation was reduced, and cell migration restrained. According to these findings, we conclude that CREPT promotes melanoma cell migration and actin filament/focal adhesion formation through RhoA activation.

## 4. Materials and Methods

### 4.1. Antibodies and Plasmids

Anti-RhoA (2117), anti-mDia1 (5486), anti-FilaminA (4762), anti-FAK (3285), anti-Talin-1 (4021), anti-Paxillin (12065), and anti-Tensin 2 (11990) monoclonal antibodies were purchased from Cell Signaling Technology Europe B.V. (Leiden, Rotterdam, Netherlands). Anti-ACTB antibodies (A2066 and A5441) were from Sigma-Aldrich Chemie N.V. (Zwijndrecht, South Holland, Netherlands). Anti-CREPT monoclonal antibody and plasmids (pcDNA3.1-HA-CREPT, pLL3.7-shRNA-CREPT) were kindly provided by Zhijie Chang (Tsinghua University, Beijing, China) [38].

### 4.2. Cell Lines and Culture Conditions

Human melanoma cell lines BLM, M14, Mel57, 1F6, and 530 were maintained in Dulbecco’s modified eagle’s medium (DMEM) supplemented with 10% fetal bovine serum (FBS). Normal human epidermal neonatal melanocytes (NHEMs) were cultured with Melanocyte Growth Medium-4 BulletKit (Lonza Benelux BV, Breda, Netherlands). All cell lines above were routinely maintained in 5% CO_2_ at 37 °C. Immortalized human melanocytes (hmels) were grown in RPMI medium supplemented with 10% FBS, tetradecanoyl phorbol acetate (TPA, 200 nM), cholera toxin (200 pM), human stem cell factor (SCF, 10 ng/mL), and endothelin 1 (10 nM), and cultured under conditions of 10% CO_2_ at 37 °C.

### 4.3. Reverse Transcription Polymerase Chain Reaction (RT-PCR) and Quantitative PCR

Total RNA was extracted with TRIzol reagent (Invitrogen, Carlsbad, CA, USA). Reverse transcription was performed with 1 μg of total RNA using the Superscript III First-Strand Synthesis System (Invitrogen). In total, 50 ng of cDNA were used for each reaction. The sequences of PCR primers were as follows:

CREPT forward: 5’-TAT AGG TAC CAT GTC CTC CTT CTC TGA G-3’,
CREPT reverse: 5’-TAT ACT CGA GCT AGT CAG TTG AAA ACA GGT C-3’;
ACTB forward: 5’- GTC ATT CCA AAT ATG AGA TGC GT-3’,
ACTB reverse: 5’- AAT GCT ATC ACC TCC CCT GT-3’.

Quantitative PCR was performed in duplicate, using the iCycler iQ5 platform (Bio-Rad Laboratories, Munich, Germany) with specific TaqMan Gene Expression assays (Applied Biosystems, Foster City, CA, USA). Gene expression levels are presented as relative ratio.

### 4.4. Western Blotting

Cells were washed with ice-cold phosphate-buffered saline (PBS) and lysed in lysis buffer (50 mM Tris-HCl pH 7.4, 150 mM NaCl, 1 mM ethylenediaminetetraacetic acid (EDTA), 1% NP-40, protease inhibitor cocktail and phosphatase inhibitor cocktail) for 30 min on ice. Lysates were cleared by centrifugation at 12,000× *g* for 10 min at 4 °C. Samples were electrophoresed and transferred to polyvinylidene difluoride (PVDF) membranes. Blocking was conducted in Odyssey blocking buffer (LI-COR Biosciences, Lincoln, NE, USA) for 1 h at room temperature, followed by overnight incubation of diluted primary antibodies. Membranes were further incubated with IRDye-labeled secondary antibodies (LI-COR) for 1 h at room temperature and scanned using Odyssey Infrared Imaging System (LI-COR).

### 4.5. Cell Transfection

The transfection was performed with DharmaFECT kb DNA transfection reagent (Horizon Discovery Group, Waterbeach, United Kingdom) according to the manufacturer’s instructions. Melanoma cells were transfected with pcDNA3.1-HA-CREPT for CREPT overexpression and pcDNA3.1-HA as the control. Stable clones were selected using 600 µg/mL G418 (Thermo Fisher Scientific, Waltham, MA, USA) and maintained in culture medium with 300 µg/mL G418. CREPT-depleted cells were generated by transfection with plasmids (pLL3.7-shRNA-CREPT: pLKO.1-puro = 10:1) and control cells were transfected with plasmids (pLL3.7-shRNA-control: pLKO.1-puro = 10:1). Stable cells were selected based on florescence and maintained in culture medium with 1 µg/mL puromycin (Thermo Fisher Scientific, Waltham, MA, USA). Western blotting was performed to detect the transfection efficiency. BLM cells with stable transfection were used in the following experiments.

### 4.6. Cell Proliferation Assay

Transfected cells were seeded in a 96-well plate at a density of 2000 cells/well and allowed to attach for 6 h. After attachment, cells were fixed with 10% trichloroacetic acid at 0, 24, 48, 72, and 96 h, followed by staining with sulphophodamine B (SRB) as described [39]. The absorbance was measured at 510 nm using a microplate reader (Victor 1420, Wallac, Turku, Finland). All proliferation data were normalized to the 0-h time point and showed as the percentage growth rate.

### 4.7. Colony Formation Assay

A total of 500 cells/well were seeded in a 6-well plate with culture medium. After 10 to 14 days, cells were fixed with methanol for 15 min and stained with 0.1% crystal violet for 20 min. Plates were washed and air-dried. Three independent experiments were performed in 6-well plates. Colonies counting was performed by ImageJ with a minimum size of a 10-pixel area.

### 4.8. Ring-Barrier Migration Assay

Cell migration was observed using the ring-barrier migration assay as previously described [40]. Briefly, 3 × 10^5^ cells were seeded on fibronectin (10 μg/mL, Sigma-Aldrich, St. Louis, MO, USA)-coated coverslips in an Attofluor Cell Chamber (Invitrogen, Carlsbad, CA, USA) and migration was monitored for 24 h in DMEM medium supplemented with 1% FBS. Time-lapse imaging was conducted on an Axiovert 100 M inverted microscope (Carl Zeiss B.V., Sliedrecht, Netherlands). Parameters of cell migration were obtained from the acquired sequence using AxioVision SE64 Rel.4.9.1 software (Carl Zeiss, Oberkochen, Germany). Three independent experiments were performed in triplicates, and from each replicate, 10 cells’ trajectories were obtained.

### 4.9. 3D Invasion Assay

Cytodex-3 microcarrier beads (Sigma-Aldrich, St. Louis, MO, USA) were mixed with 5 × 10^5^ cells in a falcon tube and incubated at 37 °C for 6 h with gentle mixing to ensure complete coverage of the beads. Next, the suspension was transferred to a 25-cm^2^ culture flask and incubated overnight to remove unattached cells. The cell coated beads were embedded in 1.6 mg/mL collagen gel in a 24-well plate. Post-polymerization at 37 °C for 30 min, the gel was covered with 500 μL of culture medium. Cell invasion was followed for up to 5 days with a 10× (NA 0.30) objective lens on an Axiovert 100 M microscope (Carl Zeiss, Oberkochen, Germany). Image acquisition and analysis were conducted with AxioVision software (Carl Zeiss, Oberkochen, Germany). For each cell line, images of 10 to 15 beads were captured to measure the dispersion distance and the assay was performed three times independently.

### 4.10. Gene Expression Profiling

RNAs isolated from cell lines were processed with the Illumina TruSeq Stranded mRNA Library Prep Kit (Illumina, San Diego, CA, USA). The resulting indexed DNA libraries were pooled and sequenced according to the Illumina TruSeq Rapi v2 protocol on an Illumina HiSeq2500 sequencer using 50 base-pair reads. Adapter sequences were trimmed off, and resulting sequences were mapped against the GRCh38 human reference sequence using HiSat2 (version 2.1.0). Transcripts were quantified using htseq-count (version 0.9.1). Differential gene expression was performed based on the cutoff criteria of a 1.5-fold change and *p*-value < 0.01. Gene ontology and pathway analysis were performed with Ingenuity Pathway Analysis (IPA, Qiagen, Hilden, Germany) and DAVID bioinformatics resources 6.8 [41]. Heat maps were created by Heatmapper [42] with variance stabilizing transformed values from DESeq2 package.

### 4.11. Immunofluorescence Staining

Cells were cultured on fibronectin (10 μg/mL)-coated 12-mm glass coverslips in 24-well plates. After incubation overnight, cells were fixed with 4% paraformaldehyde in PBS for 15 min, and permeabilized with PBS/0.1% Triton X-100 for 5 min. After being blocked with 1% BSA for 1 h at room temperature, cells were incubated with Rhodamine Phalloidin (Cytoskeleton Inc, Denver, CO, USA) and Anti-Vinculin Alexa Fluor 488 (Invitrogen, Carlsbad, CA, USA) for 1 h at room temperature. Nuclei were stained with 4’,6-diamidino-2-phenylindole (DAPI, Invitrogen, Carlsbad, CA, USA). Fluorescent images were acquired using a Zeiss LSM 510 META microscope with a 63× lens (NA 1.40, Carl Zeiss, Oberkochen, Germany). F-actin quantification was performed using LPIXEL ImageJ Plugins (LPIXEL Inc, Tokyo, Japan), and focal contacts were quantified in Fiji as described [43,44].

### 4.12. Small GTPase Activation Assay

The RhoA activation level of each cell line was evaluated using the RhoA G-LISA Activation Assay kit (Cytoskeleton Inc, Denver, CO, USA) according to the manufacturer’s instructions.

### 4.13. Inactivation of Rho

BLM cells were grown to 30% to 50% confluence in 25 cm^2^ flasks under normal culture conditions. Culture medium was aspirated, and cells were treated with 1 μg/mL C3 transferase (Cytoskeleton Inc, Denver, CO, USA) diluted in serum-free DMEM. After incubating for 2 h at 37 °C, the inactivation efficiency was measured using the small GTPase activation assay. Treated cells were fixed for immunofluorescence staining or collected for migration assay as mentioned above.

### 4.14. Statistical Analysis

Statistical analysis was performed using GraphPad Prism version 5.01 (GraphPad Software, San Diego, CA, USA). Multiple groups were compared using one-way ANOVA followed with Dunnett’s multiple comparison test. For the comparison of two groups, student’s *t*-test was performed for normally distributed data sets; otherwise, the Mann–Whitney test was executed. Time-varying covariates comparisons were evaluated by two-way ANOVA. A significant difference was considered when *p* < 0.05.

## 5. Conclusions

This study demonstrated the oncogenic role of CREPT in melanoma by promoting cell proliferation, migration, and invasion. This phenomenon is dependent on the upregulation and activation of RhoA-induced actin organization and focal adhesion assembly. Our findings contribute to a better understanding of melanoma progression, and provide a potential therapeutic target for melanoma treatment.

## Supplementary Materials

The following are available online at https://www.mdpi.com/2072-6694/12/1/33/s1, Figure S1: CREPT expression examined by western blotting., Figure S2: CREPT affects M14 and MEL57 proliferation, migration and invasion. CREPT was overexpressed in M14 cells and knocked down in MEL57 cells, Figure S3.1: Protein expression of RhoA, mDia1, FilaminA and FAK by western blotting in CREPT modified BLM cells. Red bands show ACTB as loading control. Intensity ratios of each band is displayed in tables next to the images. The ratios were calculated on the basis of the sample with the ratio of 1, Figure S3.2: Protein expression of Talin-1, Paxillin and Tensin2 by western blotting in CREPT modified BLM cells. Red bands show ACTB as loading control. Intensity ratios of each band is displayed in tables next to the images. The ratios were calculated on the basis of the sample with the ratio of 1.
