# Supplementary material for: CREPT Promotes Melanoma Progression Through Accelerated Proliferation and Enhanced Migration by RhoA-Mediated Actin Filaments and Focal Adhesion Formation
Source: Cancers (Basel). 2019 Dec 20;12(1):33. doi: 10.3390/cancers12010033 (PMC7016535; doi:10.3390/cancers12010033)
Supplement: Supplementary file 1 [file cancers-12-00033-s001.pdf]

Article

CREPT Promotes Melanoma Progression Through Accelerated Proliferation and Enhanced Migration by Rhoa-Mediated Actin Filaments and Focal Adhesion Formation

Hui Liu, Ann L.B. Seynhaeve, Rutger W.W. Brouwer, Wilfred F.J. van IJcken, Liu Yang, Yinyin Wang, Zhijie Chang * and Timo L.M. ten Hagen *

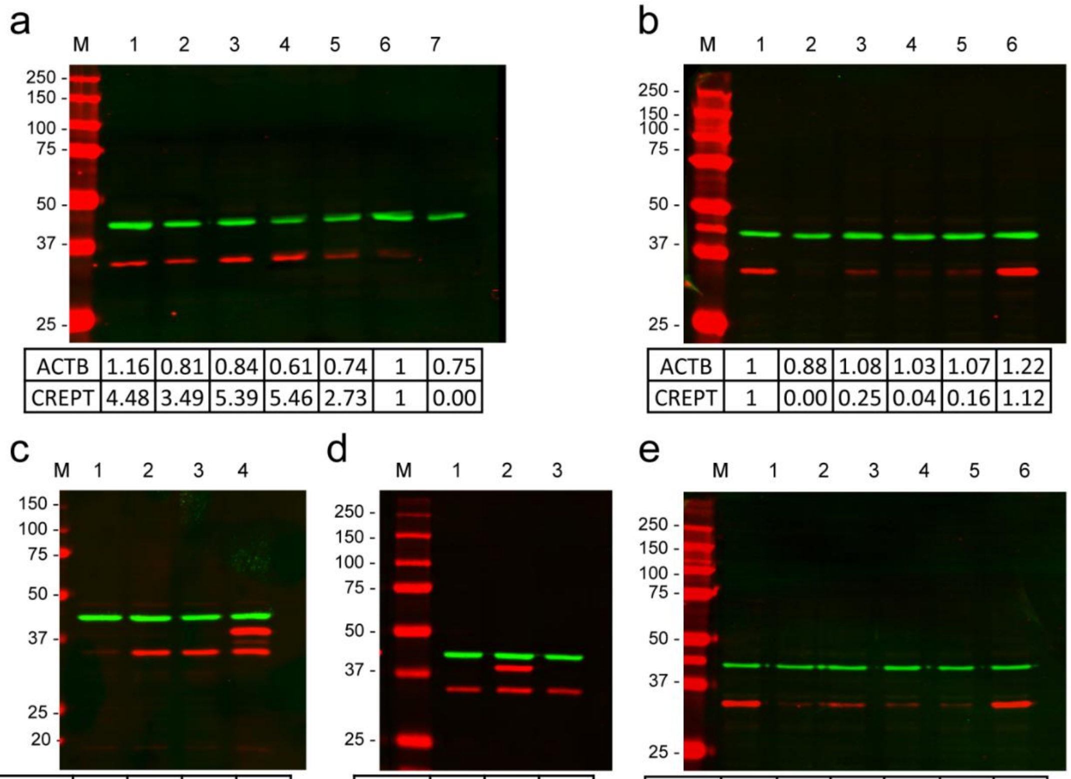

(a)

ACTB	1.16	0.81	0.84	0.61	0.74	1	0.75
CREPT	4.48	3.49	5.39	5.46	2.73	1	0.00

(b)

ACTB	1	0.88	1.08	1.03	1.07	1.22
CREPT	1	0.00	0.25	0.04	0.16	1.12

(c)

ACTB	1.06	1	0.83	1.01
CREPT	0.16	1	1.01	2.08

(d)

ACTB	1	1.07	0.87
CREPT	1	1.98	0.92

(e)

ACTB	1	0.95	1.18	1.08	0.92	1.04
CREPT	1	0.10	0.43	0.21	0.12	1.07

Figure S1. CREPT expression examined by western blotting. green: ACTB; red: CREPT. Intensity ratios of each band is displayed below images in tables. The ratios were calculated on the basis of the sample with the ratio of 1. (**a**) CREPT in melanoma cells and melanocytes. M: marker; 1: BLM; 2: M14; 3: MEL57; 4: 1F6; 5: 530; 6: hmel; 7: NHEM. (**b**) BLM cells were transfected with shRNAs to knockdown CREPT expression. M: marker; 1: scrambled shRNA; 2: shRNA-1; 3: shRNA-2; 4: shRNA-3; 5: shRNA-4; 6: BLM. (**c**) CREPT knockdown and overexpression in BLM cells used in the main text. M: marker; 1: shRNA-1; 2: scrambled shRNA; 3: empty vector; 4: vector + CREPT. (**d**) CREPT was overexpressed in M14 cells. M: marker; 1: empty vector; 2: vector + CREPT; 3: M14. (**e**) MEL57 cells were transfected with shRNAs to knockdown CREPT expression. M: marker; 1: scrambled RNA; 2: shRNA-1; 3: shRNA-2; 4: shRNA-3; 5: shRNA-4; 6: MEL57.

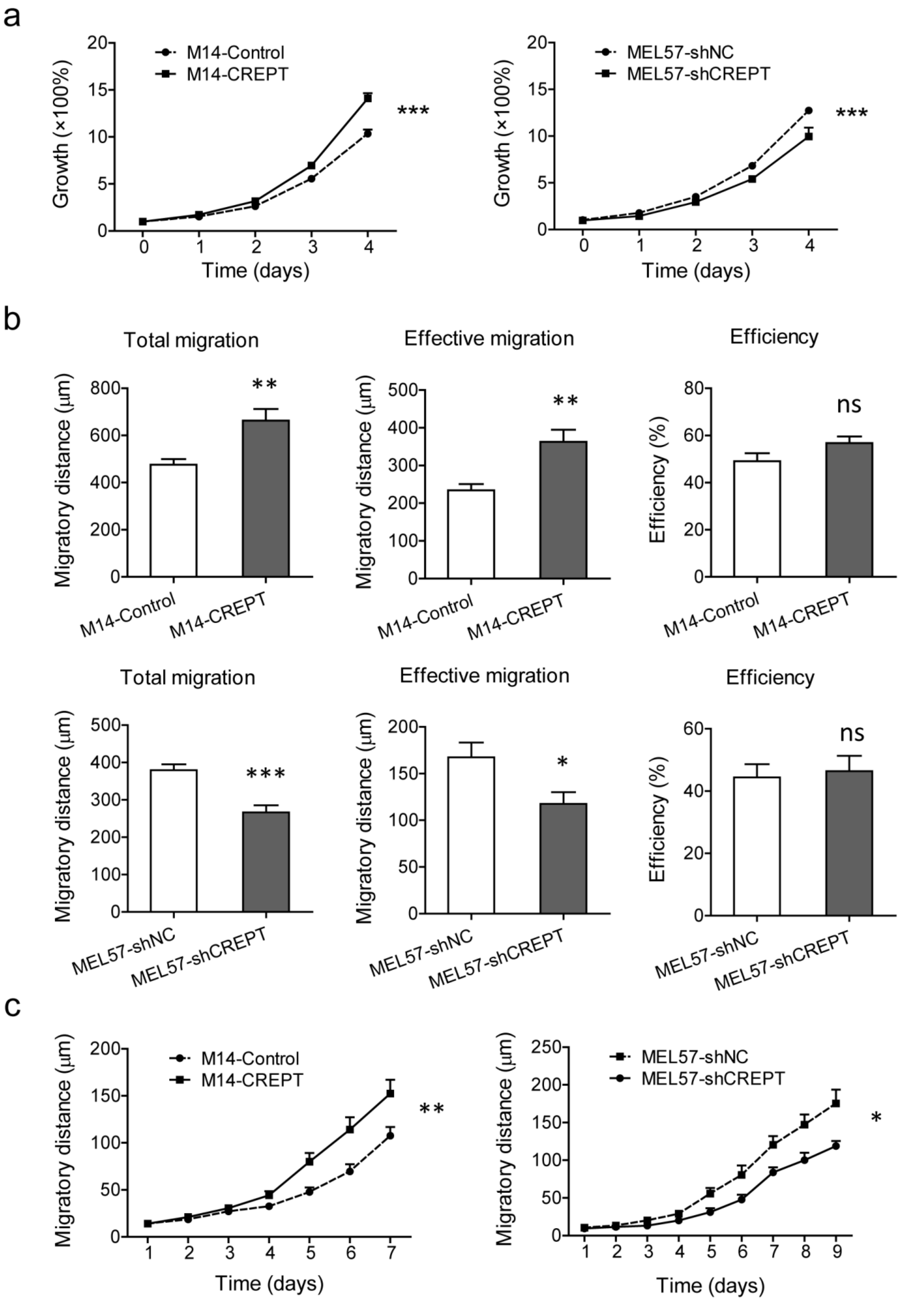

Figure S2. CREPT affects M14 and MEL57 proliferation, migration and invasion. CREPT was overexpressed in M14 cells and knocked down in MEL57 cells. (**a**) Cell growth rate was examined by SRB assay and standardized to the first time point. (**b**) Migratory parameters was obtained from ring-barrier migration assay by tracking at least thirty cells. (**c**) Cell invasive capacity was evaluated through microcarrier based spheroid 3D invasion assay. Cell dispersion distances were measured over time. Data represent mean ± SEM of three independent experiments * $p < 0.05$, ** $p < 0.01$, *** $p < 0.001$. ns: not significant.

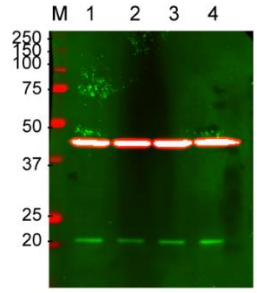

	1	2	3	4
	BLM-shNC	BLM-shCREPT	BLM-Control	BLM-CREPT
RhoA (21kDa)	1.15	0.72	1	1.23

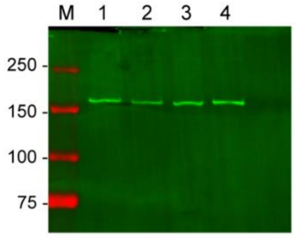

	1	2	3	4
	BLM-shNC	BLM-shCREPT	BLM-Control	BLM-CREPT
mDia1 (150kDa)	1.03	0.81	1	1.20

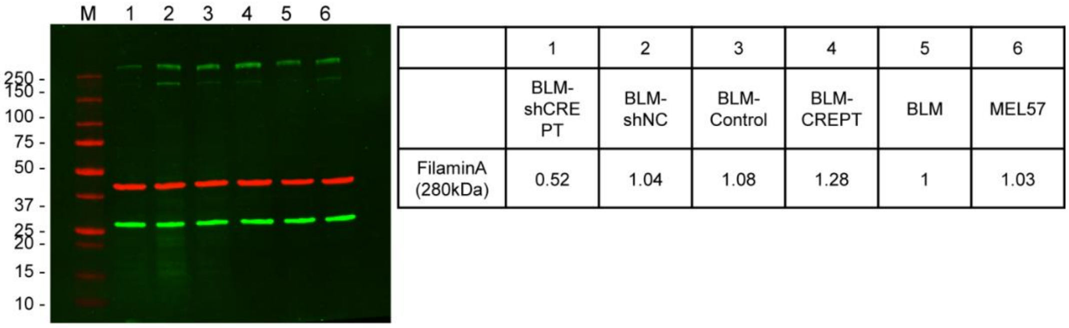

	1	2	3	4	5	6
	BLM-shCREPT	BLM-shNC	BLM-Control	BLM-CREPT	BLM	MEL57
FilaminA (280kDa)	0.52	1.04	1.08	1.28	1	1.03

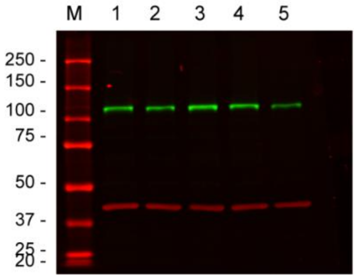

	1	2	3	4	5
	BLM	BLM-Control	BLM-CREPT	BLM-shNC	BLM-shCREPT
FAK (125kDa)	1	0.91	1.31	1.11	0.57

Figure S3.1. Protein expression of RhoA, mDia1, FilaminA and FAK by western blotting in CREPT modified BLM cells. Red bands show ACTB as loading control. Intensity ratios of each band is displayed in tables next to the images. The ratios were calculated on the basis of the sample with the ratio of 1.

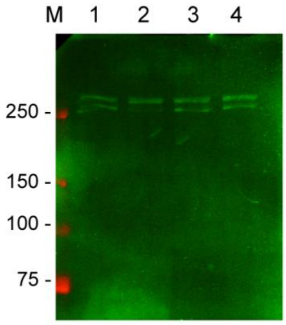

	1	2	3	4
	BLM-shNC	BLM-shCREPT	BLM-Control	BLM-CREPT
Talin-1 (270kDa)	1	0.68	1.07	1.63

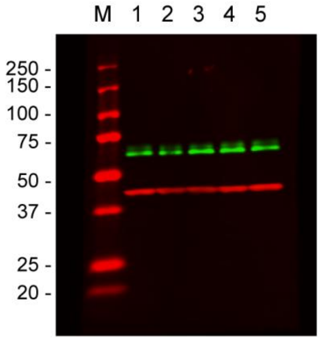

	1	2	3	4	5
	BLM	BLM-Control	BLM-CREPT	BLM-shNC	BLM-shCREPT
Paxillin (62kDa)	1	0.91	1.31	1.11	0.57

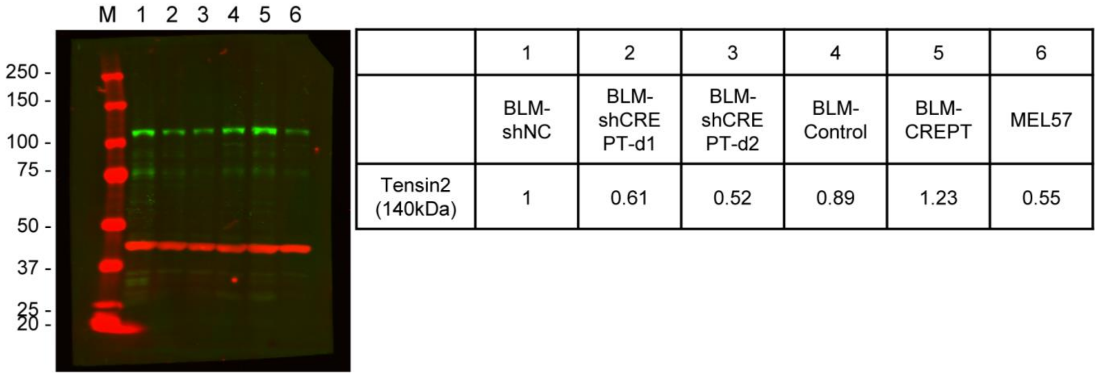

	1	2	3	4	5	6
	BLM-shNC	BLM-shCREPT-d1	BLM-shCREPT-d2	BLM-Control	BLM-CREPT	MEL57
Tensin2 (140kDa)	1	0.61	0.52	0.89	1.23	0.55

Figure S3.2. Protein expression of Talin-1, Paxillin and Tensin2 by western blotting in CREPT modified BLM cells. Red bands show ACTB as loading control. Intensity ratios of each band is displayed in tables next to the images. The ratios were calculated on the basis of the sample with the ratio of 1.

© 2020 by the authors. Licensee MDPI, Basel, Switzerland. This article is an open access article distributed under the terms and conditions of the Creative Commons Attribution (CC BY) license (http://creativecommons.org/licenses/by/4.0/).
